# Analysis of role of rat cerebral pericytes in cerebral vasospasm after subarachnoid hemorrhage and molecular mechanism of neurovascular injury

**DOI:** 10.1080/21655979.2021.1947630

**Published:** 2021-07-21

**Authors:** Zhenxing Yan, Yang Zou, Yiting Deng, Siqin Liu, Kaifeng Li, Juan Yang, Xihua Guo, Rongni He, Wenxia Zheng, Huifang Xie

**Affiliations:** a Department of Neurology, Zhujiang Hospital, Southern Medical University, Guangzhou, China; b Department of Neurology, Second School of Clinical Medicine, Southern Medical University, Guangzhou, China; c Department of Neurology, Shunde Hospital, Southern Medical University, Foshan, China

**Keywords:** Pericytes, SAH, microvascular spasm, neurovascular injury, action mechanism

## Abstract

To investigate mechanism of pericytes in the early stage of subarachnoid haemorrhage (SAH) and its associated microvascular spasm and neurovascular injury, 100 healthy 8-week-old Sprague-Dawley male rats were taken as subjects and divided into four groups: group A (sham operation, control group), group B (SAH operation group), group C (SAH operation group treated with scutellarin), and group D (SAH operation group treated with L-nitro-arginine). 72 hours after the operation, the rats were conducted assessment of neurological impairment, observation of microangiography, detection of blood-brain barrier permeability, observation of skull base haemorrhage, identification of pericyte culture, and measurement of blood nitric oxide. The results showed that neurological impairment score, degree of micro-vasoconstriction, and BBB permeability of group C were significantly better than those of group B and D (P<0.05), there was no significant difference between group C and group A (P>0.05). There were significantly fewer blood clots in the brain of group C, and the order of expression levels of α-smooth muscle actin (α-SMA) in perioperative cells of the four groups from highest to lowest were D, B, C, and A. Nitric oxide concentration inhibited expression of α-SMA in pericytes after SAH at both protein and mRNA levels. The detection results of nitric oxide in the blood of four groups of rats confirmed that pericyte phenotype conversion and actin α-SMA expression could be prevented by upregulation of nitric oxide in serum, so as to relieve pathological symptoms after SAH operation.

## Graphical abstract

**Figure:**
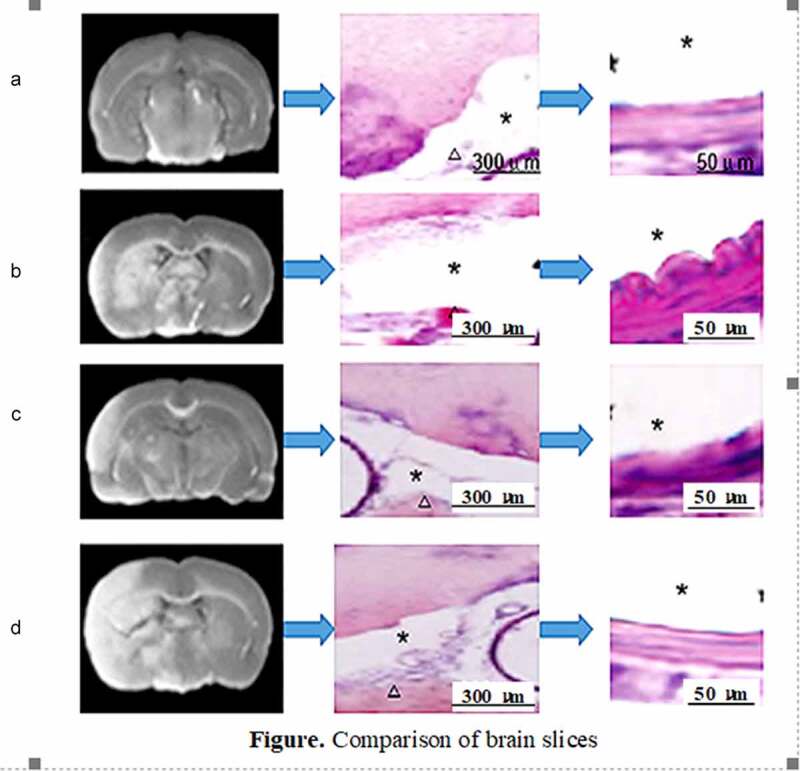

## 1. Introduction

Subarachnoid hemorrhage (SAH) is usually attributed to brain diseases, such as rupture of blood vessels on the surface of the brain or intracranial aneurysms, rupture of the epidural membrane, and cerebrovascular deformities and so on. It may cause blood to flow into the subarachnoid space and may damage the central nervous system [1]. It is a nervous system emergency that accounts for 5% of strokes. After SAH occurs, due to some complicated pathological characteristics, intracranial pressure decreases and cerebral blood flow decreases suddenly in the early stage, along with microvasospasm, circulatory system disorder, and blood–brain barrier damage [2]. Studies found that about one-quarter of SAH patients died, and half of the surviving patients had neurological damage. Cerebral vasospasm (CVS) is a common high-risk complication in SAH. Once it occurs, it often causes severe local brain tissue ischemia and even cerebral infarction, and it has become the main cause of death and disability in SAH [3].

In recent years, there has been a lot of research on SAH, mainly including free radicals, reduction of NO, and a series of mechanisms such as inflammation. NO is a kind of gaseous-free radical, which is widely present in many kinds of cells including vascular endothelial cells, and plays an important role in tissue generation, metabolism, and apoptosis [4]. SAH not only reduces the NO content, but also reduces the responsiveness of vascular smooth muscle to NO, which in turn causes the blood vessels to fail to maintain their normal diastolic function, leading to vasospasm. Under pathologic conditions, NO participates in inflammatory response through its neuromodulation and causes nerve damage. Pericytes are cells whose microvascular walls are located outside the endothelial spleen cells and surround the endothelium. The relationship between it and the cells plays an important role in the regulation of blood vessel growth. Some researchers observed that α-smooth actin (α-SMA) can be expressed in granulation tissue and breast cancer interstitial neovascularization through immunohistochemistry and electron microscopy observation methods [5]. Bandopadhyay and Nakanod found that pericytes can selectively respond to smooth muscle subtype actin. These results suggest that smooth muscle actin may serve as a specific marker of pericytes.

Cerebral vasospasm is the main reason for the poor prognosis of subarachnoid hemorrhage. The pathogenesis of cerebral vasospasm is extremely complicated. However, many scholars believed that hemoglobin was caused by the inhibition of endothelial cell-mediated vasodilation. Due to insufficient blood supply to the brain caused by CVS, the permeability of the BBB increased, secondary cerebral ischemia damage, and even permanent neurological defects [6]. At present, there is no specific therapy to prevent CVS. However, in recent years, with the in-depth exploration of vascular endothelial cells, novel ways to effectively prevent CVS are found. Studies found that the use of astragaloside IV can alleviate CVS after SAH in rats by interfering with the inflammatory signal pathway mediated by TLR4NF-kB. There was also the adoption of L-arginine in rabbit SAH. Through detections of the amount of brain tissue water, Na^+^, K^+^, Ca^2+^, and morphological observations, it was found that L-arginine can significantly reduce the diameter and cross-sectional area of the cerebral basal artery, which also can significantly reduce cerebral edema and effectively prevent cerebral vasospasm [7]. However, there were relatively few reports about the mechanism of action of L-nitro amino acids and baicalein after CVS intervention in rats after SAH, and the changes in NO concentration in vivo. In this study, the molecular mechanism of neurovascular injury was explored by measuring the neurological deficit score, the degree of micro-vasoconstriction, the BBB permeability, and morphological observation in rats, so as to provide experimental basis for the clinical treatment of cerebral vasospasm after SAH.

## 2. Materials and methods

### 2.1 Experimental animals and materials

A total of 100 SPF healthy Sprague-Dawley male rats weighing about 300 g were selected at 8 weeks of age. The rats were divided into four groups with 25 in each group. After numbered, the rats were kept in cages under the same environmental conditions, with a constant temperature of 25°C and good ventilation, and the rats were guaranteed to drink and eat freely. The day before the puncture, fast and no water were conducted to them for three hours. All animal procedures in this test were approved by the Laboratory Animal Control Committee, and the experimental methods were carried out in accordance with the approval guidelines.

### 2.2 Construction of subarachnoid hemorrhage model

Three groups of rats were first injected with 1.25% pentobarbital solution of 55 mg/kg for anesthesia treatment, after which the body was fixed on the operating table supine and neck hair was removed after disinfection, the incision was about 2.5 cm, and the fat and muscle tissue were obtuse to separate. Carotid artery separation was performed under the microscope, the small branches of external carotid artery and occipital artery were cut off by electrocoagulation, and the internal carotid artery and common carotid artery were clipped with arterial clamp. The external carotid artery was then ligated and a small opening was made at the distal end, through which a 5–0 nylon thread about 6 cm long was inserted, and the ligation was performed with a noose to block blood flow. The artery clamp was open, the positions of the internal and external carotid arteries were adjusted, the nylon line was penetrated into the internal carotid artery again, the length of the line about 1 cm was kept out, and the rats’ breathing was observed for a while. Finally, the thread plug was removed and the incision was closed, and the other group was treated with the same procedure without perforating their arteries. The original feeding conditions were still maintained after the operation. For the rats with extremely severe symptoms, low-dose atropine could be injected to relieve the great vasospasm and minimize the death.

Group A was sham operation group and taken for control; group B was the operation group, which only performed puncture of internal arterial plug without drug treatment; group C was treated with operation and drugs, after the internal artery puncture operation, scutellarin solution with a purity of more than 99% and a concentration of 0.25 mg/kg was injected by intraperitoneal injection; and group D was treated with operation and drugs, after the internal artery puncture operation, L-nitro-arginine solution with a purity of more than 99% and a concentration of 0.25 mg/kg was injected by intraperitoneal injection

### 2.3 Neurologic function assessment

Since the evaluation of rat neural function adopting the enhanced Zeal Longa five-point scale mainly relied on the subjective judgment of the experimentalist, the content of the experiment should be kept secret to the grader in advance, so as to ensure the objectivity of the evaluation results. The whole process was evaluated for 4 times, namely after SAH operation, 12, 24, and 72 hours after operation, the free activity of rats should be observed for more than 5 minutes each time, and the evaluation criteria were shown in Table 1.

**Table 1.** Zeal-Longa five-point scale scoring method

Score	Evaluation standard
0	The limbs are bilaterally symmetrical and behave normally, and respond quickly when touching the body without obvious neurological deficits.
1	Relatively little activity, the limb activity shows a slight bilateral asymmetry, the response is not rapid when touching the body, and the neurological deficit is not obvious.
2	Less activity, limb activity shows bilateral asymmetry, the reaction is slow when touching the body, and the neurological deficit is more obvious.
3	Very little activity, limb activity shows obvious bilateral asymmetry, unresponsiveness when touching the body, and obvious neurological deficits.
4	Almost no activity, almost no response to touch, neurological deficit.

### 2.4 In vivo fluorescence imaging

## I. Microangiography

The femoral artery was exposed after SAH operation, and 2% Evans Blue (EB) solution (produced by Sigma Company) was punctured and injected. The imaging of arterial branch vessels was observed under fluorescence imaging machine 1 h later, and the above steps were repeated 24 hours later to observe the changes of microvessels.

## II. BBB permeability test

In order to facilitate subsequent experiments, five rats were randomly selected from each group 24 hours after operation for in vivo femoral vein injection of EB with concentration of 85 mg/kg. Brain tissue was stripped and put into a fluorescence imager (emitting light wavelength was about 680 nm, excitation light wavelength was about 470 nm) to collect images, after which the brain tissue was weighed, dimethylformamide was added, and the supernatant was taken after a 48 h water bath at 37.5°C and centrifuged at 3000 r/min for 10 min. The absorbance was determined and the EB content in the sample tissue was calculated according to the standard EB spectrophotometry curve.

### 2.5 Pathological observation of brain and preparation of coronal section of brain tissue

I. After the successful construction of the SAN model and in vivo observation, the rats were sterilized and sacrificed, and the brain pathology was observed. The rats were graded one by one according to the skull base hemorrhage condition. The score was 0–3, 0 indicated no subarachnoid hemorrhage, 1 indicated subarachnoid hemorrhage; 2 indicated partial blood vessels with obvious blood clots; 3 indicated large number of blood clots appeared in most of the blood vessels.

II. Brain tissue was fixed in paraformaldehyde solution at low temperature for 24 h, and then dehydrated with paraformaldehyde-sucrose solution. After brain tissue sank to the bottom, OCT embedding treatment was performed, and slices were stored at minus 20°C. After reheating, phosphate buffer salt solution was used and kept for 5 min each time. The methanol solution was used for sealing treatment for about 30 min, and the previous step was repeated. Bovine serum albumin was adopted to close the slices and they were incubated for 30 min. The primary antibody was added and incubated for 2 h at room temperature and 12 h at low temperature. After the slices were rinsed again, sheep serum protein was added and cultured in dark for 80 min. Washed, then rinsed with pure water, the slices were sealed and stained with DAB.

### 2.6 Construction of culture system of pericytes in vitro

In order to verify whether the phenotypic transformation of pericytes was caused by changes in nitric oxide concentration, the expression of pericytes in brain tissue of healthy mice should be observed in vitro. After taking rat brain tissue and placing it in cryogenic MEM/BESS solution (purchased from Shanghai Beinuo Biotechnology Co., LTD., produced by Hyclone Company), the olfactory bulb, cerebellum, and great vessels of the frontal lobe of the brain were taken out, and the remaining brain tissue was broken, mixed with MEM/BESS solution, and centrifuged at 1200 r/min for about 6 min. The supernatant was removed, and the digestion solution was added for more than 1 hour at a constant temperature of 37.5°C. After that, fetal bovine serum was added to terminate the digestion, the cells were suspended by shaking and centrifuged at 4000 r/min for 10 minutes, then the underlying cells were resuspended in ECGM-1 medium (produced by ZenBio). Subculture was carried out at 37.5°C, humidity was 95.5%, carbon dioxide was 5%, and the cell identification could be carried out in the third generation.

### 2.7 Western blot detection

Cultured pericytes were mixed with RIPA lysate, lysed at 4°C for 30 min, centrifuged at 12,000 r/min for 5 min, and the histone proteins were extracted. After the polypropylene gel was prepared, with 20 μL sample in each hole, three parallel samples were set for each sample. Protein electrophoresis was carried out with glycine buffer solution, 85 V constant pressure electrophoresis was used for gluing for 20 min, and 110 V constant pressure electrophoresis for gluing for 80 min. At a low temperature of 5°C, the membrane was transferred at a constant current of 100 mA. PBST buffer was adopted to rinse them three times for more than 15 min each, and incubated in a shaker for 2 h at room temperature. Antigen antibody reaction was performed, primary antibody was α-SMA (1:2500) and GAPDH (1:1000), and PEST rinsing was repeated three times for each antibody addition. Development, imaging, and analysis of strip optical density value were conducted in turn, and the protein expression content was calculated.

### 2.8 RT-PCR detection

The cultured pericytes were mixed with TRIZOL lysate and left at room temperature for 10 min to separate the protein and nucleic acid, and centrifuge at 10,000 r/min for 10 min to extract RNA. Mixed with TRIZOL lysate again, the solution was shaken well and left for 5 minutes. Centrifuged at 10,000 r/min for 15 min, the samples were separated. The intermediate layer was extracted and the RNA was extracted with IPA. After washing the precipitate and drying it sufficiently, the purity of the RNA sample was measured with an ultraviolet spectrophotometer. Reverse transcription reaction was carried out after meeting the standard (pre-denaturation at 95°C for 5 min; denaturation at 95°C for 40s; annealing at 55°C for 40s; and extension at 75°C for 40S; the whole reaction was repeated for 30 cycles). The PCR product was placed on an agarose gel and electrophoresed. Developing, image, analysis of the optical density of the band were conducted in turn, and the expression of α-SMA was calculated.

### 2.9 Detection of nitric oxide in blood of postoperative rats

The brain tissue sample prepared before was taken out, mixed with normal saline, centrifuged at 3500 r/min for 10 min. Then the supernatant was taken, mixed with Regent I and II, and put into 37.5°C water bath for 1 h; after which the regent III and IV were added, left at room temperature for 35 min, and centrifuged at 3800 r/min for 10 min; then the supernatant was added to the developer, left for 15 min, and the absorbance was measured through the standard curve equation to determine the nitric oxide content.

### 2.10. Statistical analysis

All data were expressed as mean plus or minus standard deviation; SPSS 25.0 was used for statistical analysis; and ANOVA single factor analysis of variance was used for comparison between multiple groups. All tests were conducted a two-tailed test, which was considered statistically significant when *P* < 0.05.

## 3. Results

### 3.1 Evaluation of neurologic impairment

**Figure 1.**
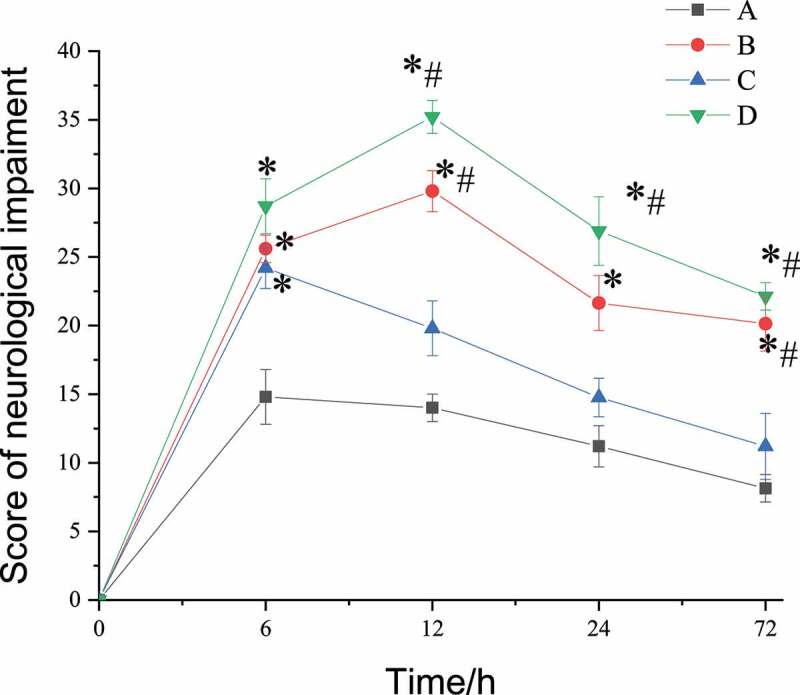
Results of evaluation of neurologic impairment

The improved Zeal Longa five-point scale scoring was adopted to evaluate the exercise and touch response of the experimental mice 6, 12, 24, and 72 hours after SAN operation. The higher the score, the more severe the neurological damage. Analysis of assessment results was shown in Figure 1. After SAH operation, there was no significant difference between the four groups. However, at the 6th hour, all four groups began to show neurological function impairments, of which group D (L-nitro-arginine treatment group) was in the worst state, while group B (SAH operation group) and group C (scutellarin treatment group) had similar functional scores and were in a better state than group D. At the 12th hour, the status of group A and C began to improve, while that of group B and D deteriorated. After 24 hours, the order of mental assessment of the four groups from good to bad was A, C, B, and D. There were significant differences between group A and the other three groups (*P* < 0.05), significant differences between group C and D (*P* < 0.05), and group B was getting better. At the 72nd hour, the neurological function scores of group A and Group C were basically stable, while those of Group B and D were still in poor state although they tended to be stable, and the scores of the four groups were significantly different from each other.

(Note: *represented a significant difference from group A (control group), *P* < 0.05; # represented a significant difference from group A and group C at the same time, *P* < 0.05.)

### 3.2 Brain microangiography results

**Figure 2.**
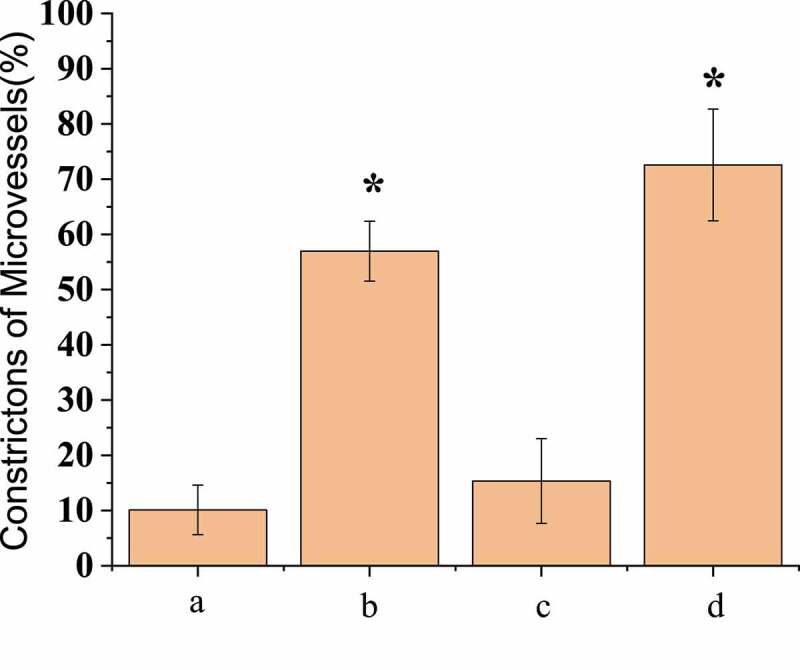
Comparison of number of micro-vasoconstrictions after operation

One hour after injecting EB into the femoral artery of the experimental rats, pear-shaped beaded contractions on the surface of the micro-vessels of the three groups of rats conducted SAH operation could be clearly observed under in vivo fluorescence microscopy, which indicated that red blood cell flow disorder was a precursor of microcirculation disorder. The micro-vasoconstriction in the surgery group was found to be different 24 hours later. The number of small vessels that were still constricted at the aortic branch was counted, and the statistical results were shown in Figure 2. It was found that the number of micro-vasoconstriction in group A, B, C, and D was 10.1% ± 4.48%, 56.96% ± 5.21%, 15.34% ± 7.68%, and 72.58% ± 10.12%, the number of micro-vasoconstriction in group C was significantly lower than that in group B and D (*P* < 0.05), and the number of vasoconstriction in group D was significantly higher than that in group B (*P* < 0.05).

(* meant that there was statistically considerable difference, *P* < 0.05.)

**Figure 3.**
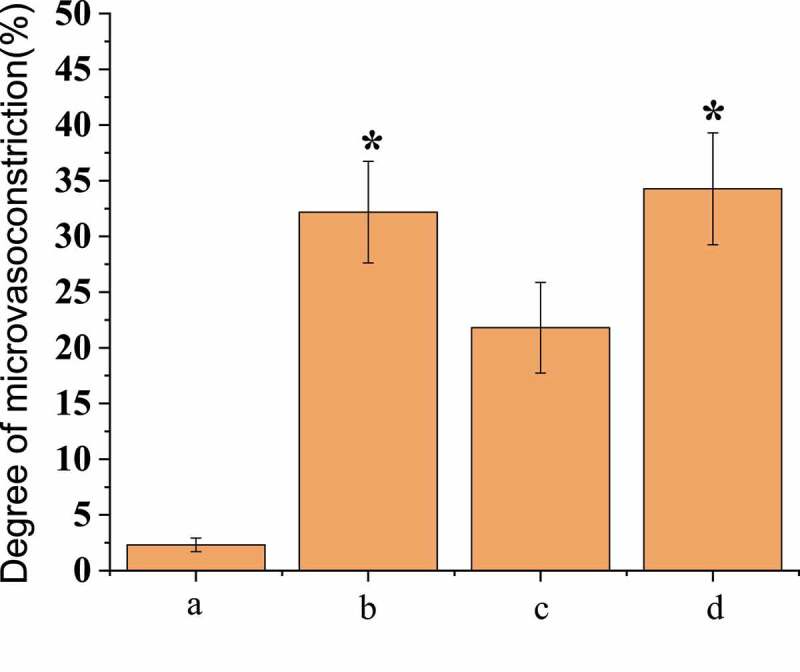
Comparison of the degree of micro-vasoconstriction after operation

Compared with the first observation, the vascular diameter of group B decreased by 32.18% ± 4.56% and that of group D decreased by 34.28% ± 5.02%, with no significant difference between group B and group D (*P* < 0.05); the vascular diameter was increased by 21.82% ± 4.07% in group C, and the vasospasm was relieved; and the vascular diameter of group A was reduced by 2.12% ± 0.61% with little change. Immunofluorescence microscopy was adopted to detect the basement membrane integrity of the micro-vessels, and the results showed that the basement membrane integrity of group B was significantly damaged compared with group A, as shown in Figure 3. In group D, the vascular basal membrane was most damaged, while in group C, the integrity of the basal membrane was between group A and group B. SAH operation rats treated with scutellarin showed improved symptoms of both microvascular spasm and blood vessel wall rupture compared with the only SAH operation groups rats.

(* meant that there was statistically considerable difference, *P* < 0.05.)

### 3.3 BBB permeability test results

**Figure 4.**
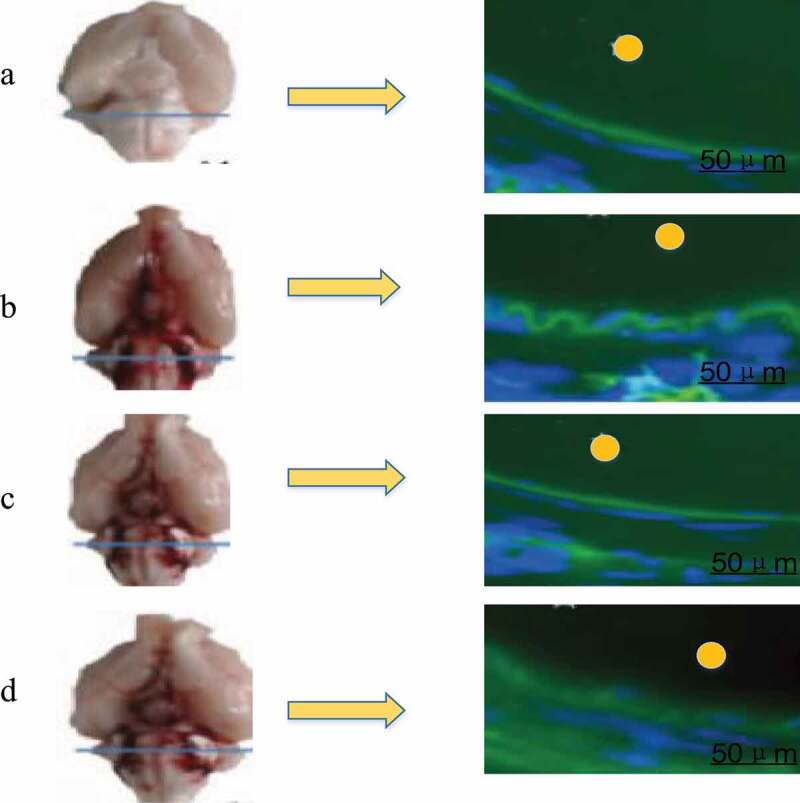
Fluorescence imaging in vivo

**Figure 5.**
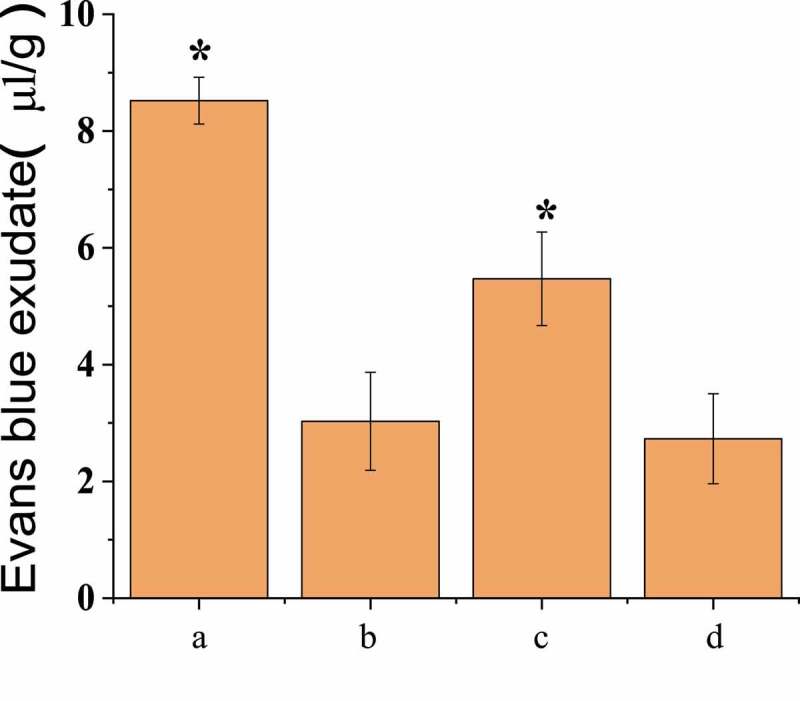
BBB permeability test results

In vivo fluorescence imaging detection showed that the infiltration amount of EB in the brain tissues of the three groups of rats conducted SAH operation was less than that of group A, as shown in Figure 4. The permeability of EB in rat brain tissue was 8.52 ± 0.40 μL/g in group A, 3.03 ± 0.84 μL/g in group B, 5.47 ± 0.81 μL/g in group C, and 2.73 ± 0.77 μL/g in group D. There was no significant difference between group A and group C, but both groups were significantly higher than group B and group D (*P* < 0.05), and there was no significant difference between group B and group D, as shown in Figure 5.

(Note: the yellow circle in the figure indicated the artery lumen.)

(* meant that there was statistically considerable difference, *P* < 0.05.)

### 3.4 Evaluation of skull base hemorrhage amount

**Figure 6.**
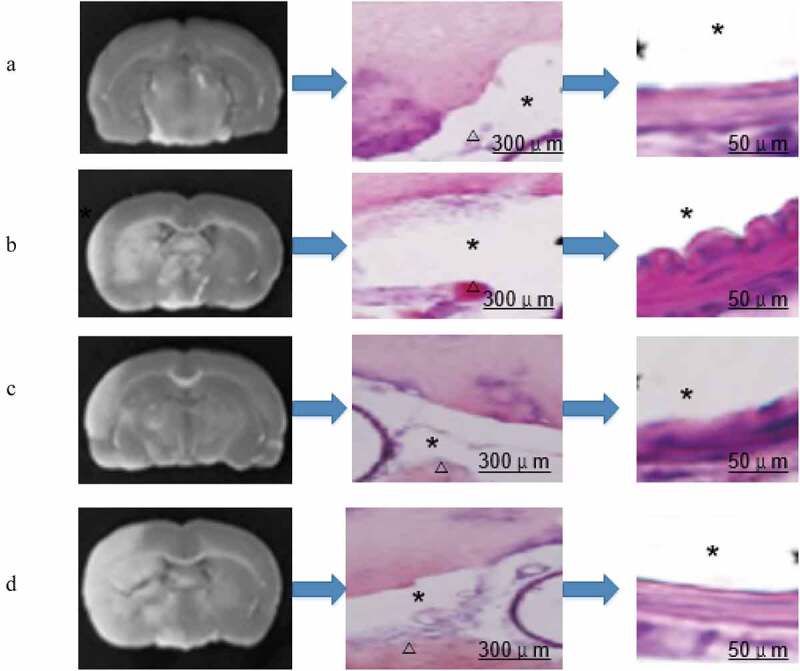
Comparison of brain slices

Pathological anatomy was conducted to observe the subarachnoid hemorrhage in the brain of the rats in the four groups. It was found that there was no hemorrhage in the skull base of the rats in group A and no clot in the arteries; group B had obvious skull base hemorrhage and obvious arterial blood clots; there was hemorrhage in skull base in group C, but it was mild, and the number of blood vessels with clot was less; and a large number of blood clots were observed in the skull base and artery vessels in group D. The scoring results were shown in Figure 6.

(Note: * in the figure represented the arterial lumen; the triangles represented blood clots in the subarachnoid space; the middle column was the general image, and the last column was the partial enlarged image.)

### 3.5 Comparison of pericyte identification results (differentiation, SMA expression, and mRNA level)

**Figure 7.**
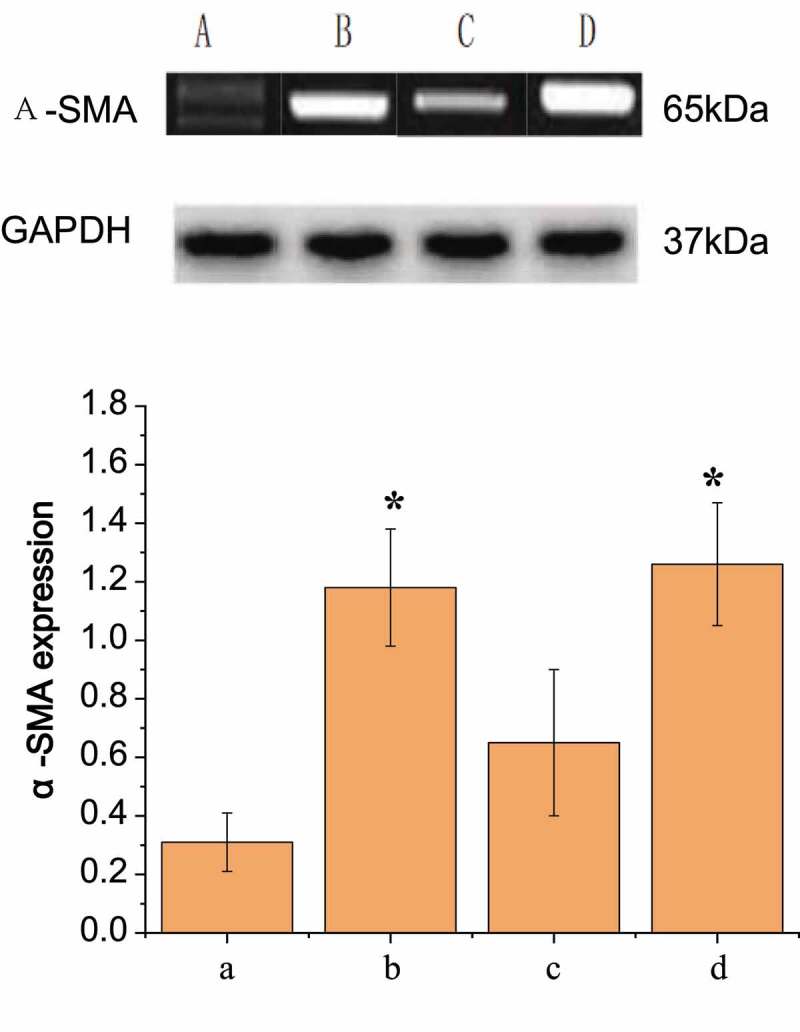
Comparison of the expression results of α-SMA in pericytes of each group

It was generally believed that α-SMA was one of the specific markers of pericytes. Pericytes were receptors for many active molecules and can express abundant contractile proteins, such as tropomyosin, myosin, and α-SMA. Western Bolt was conducted to detect the expression of α-SMA in pericytes of the four groups of rats, as shown in Figure 7. The content of α-SMA protein in group A was lower than that in group B. The pericytes in group B showed that the expression of α-SMA was up-regulated compared with that in group A (*P* < 0.05). In group C, pericytes treated with scutellarin immediately after SAH operation showed a decrease in α-SMA expression compared with that in the only SAH operation group, but still increased compared with that in the healthy control group, with no significant difference from group B (*P* > 0.05). In group D, pericytes treated with L-nitro-arginine immediately after SAH operation showed a significant increase in α-SMA expression compared with that in the only SAH operation group (*P* < 0.05).

RT-PCR detection results were shown in Figure 7, which were the same as above that the order from high to low of expression levels of α-SMA in peripheral cells of the four groups were D, B, C, and A. It was also found that the regulation of nitric oxide concentration could alleviate the induced up-regulation of pericyte α-SMA expression after SAH operation at both protein level and mRNA level.

### 3.6 Nitric oxide concentration comparison

**Figure 8.**
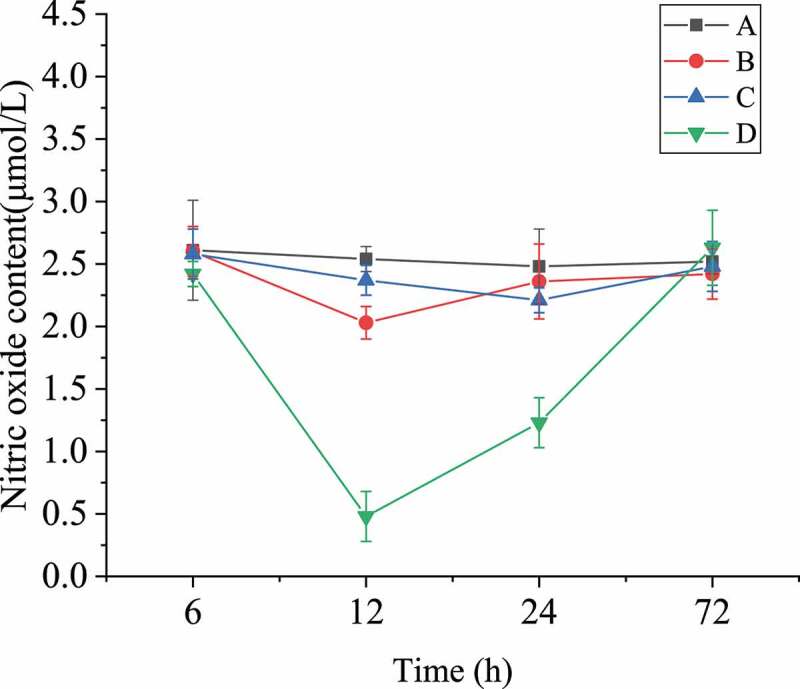
Nitric oxide content in blood of rats after SAH operation

The determination results of nitric oxide content in brain tissues of each group were shown in Figure 8. The nitric oxide content in group A remained basically stable within 72 hours, and the contents of nitric oxide in the other three groups began to decrease after surgery, and reached the lowest level at about the third hour, then gradually began to rise, and tended to be stable after 12 hours. As shown in Figure 8, the concentration of nitric oxide in group D, in which the rats were treated with L-nitro-arginine, was always lower than that of other groups, and the variation range was the largest; while that of group B was higher than group D but still lower than group A and C; the variation range of group C was small, and the content of nitric oxide was basically the same as that of group A. These results indicated that scutellarin could indeed up-regulate the content of nitric oxide in serum, and affect the phenotype transformation of pericytes and the expression of actin α-SMA, thus alleviating the pathological symptoms after SAH operation, while L-nitro-arginine had the opposite effect.

## 4. Discussion

Cerebral vasospasm is a major cause of morbidity and mortality after subarachnoid hemorrhage in modern vascular medicine. In modern medicine, it is still impossible to continuously prevent and treat cerebral vasospasm [8]. Pericytes can differentiate to stimulate the formation of new blood vessels. It has a strong effect of stimulating angiogenesis in animal models of brain injury and cerebral hypoxia. It can also express abundant contractile proteins and is a receptor for many vasoactive molecules [9]. The increase in intracranial pressure after SAH can affect the microcirculation by shrinking the perivascular cells with various substances. Studies found that pericytes can regulate endothelial cells to produce tissue-type plasminogen activator to promote coagulation [10]. Friedrich et al. (2012) [11] found that microvascular constriction and microthrombosis occurred after three hours of subarachnoid hemorrhage. Even if the cerebral blood flow was increased after SAH, the tissue couldn’t be fully perfused. In addition, it was found that the concentration of NO in the blood changed under different pathological conditions, which had opposite effects on vasoconstriction and blood circulation [12]. Yi et al. (2018) [13] mentioned that NO acted as a signal to regulate the phenotypic transformation of pericytes and thus participated in the regulation of microvessels.

The primary role of NO in cerebral blood vessels is relaxing vascular smooth muscle. The consumption of NO and the lack of its vasodilatory effect play an important role in cerebral vasospasm. After SAH occurs, the NO content decreased, and the responsiveness of vascular smooth muscle to NO may decrease, leading to the inability to maintain normal diastolic function and causing vasospasm [14]. In this study, the level of NO in Figure 8d basically recovered to the level of 6 hours after 72 hours. The restoration of NO content didn’t mean the restoration of function. Therefore, L-nitro-arginine used in group D had a certain effect. Studies revealed that arginine can relieve cerebral vasospasm. Arginine is an amino acid in the human body, which can activate guanylate cyclase through the L-arginine-NO pathway, increases the level of cyclic guanosine phosphate cGMP, and play a role in series of biological effects, such as relaxation of blood vessels, inhibition of platelet aggregation, immune regulation, and nerve conduction. Studies suggested that NO synthase existed on vascular endothelial cells, platelets, neutrophils, and brain cells. The Km value of this enzyme was extremely small, 8.4 μmol/L, which meant that L-arginine can be used to generate NO. The recovery of NO in group D also proved it. Studies showed that after the L-arginine content in endothelial cells was reduced, supplementation of exogenous L-arginine can significantly increase the synthesis and release of NO. Therefore, the mechanism by which exogenous L-arginine can significantly relieve cerebral vasospasm was synthesizing and releasing more vascular endothelial relaxing factors through vascular endothelial cells. Vascular endothelial relaxing factor can oxidize toxic hemoglobin, inhibits platelet aggregation, prevents secondary thrombosis, and scavenges oxidative free radicals [15]. Scholars supplemented more exogenous L-arginine to obtain more vascular endothelial relaxing factor, so as to improve the poor function of vascular endothelial relaxing factor caused by various reasons, and therefore maintain its biological activity. Then, the occurrence of cerebral vasospasm was effectively reduced, and the adverse consequences caused by secondary ischemic injury were prevented. Some researchers utilized L-arginine, an inhibitor of NO synthase, to expand the scope of focal cerebral ischemia. It was proved that using L-arginine can effectively improve the blood flow of focal cerebral ischemia and reduce the scope of the infarct. In this experiment, the results of group D were consistent with the results of the above research. After nitro amino acid was added, the NO content of group D decreased at 12 hours compared with 6 hours before, and increased significantly within 12–72 hours.

Some researchers found through ultrastructural observation that immunohistochemical staining can clearly indicate that cells in the walls of new blood vessels in granulation tissue were exactly marked by α-SMA. It was proved that using the cardiovascular α-SMA binding site as a marker was an effective way to study the cells surrounding the neovascularization [16]. In this work, the results were consistent with the above conclusions. The expression level of α-SMA in the peripheral blood of the four groups increased from ACBD, indicating that α-SMA was expressed in pericytes. Some studies found that the severity of cerebral vasospasm after SAH was related to inflammatory factors such as IL-6 and IL-1β. Reducing the levels of IL-6 and IL-1β downstream of the inflammatory pathway can effectively inhibit the expansion of inflammation and play a role in blood vessels. In this study, baicalein and L-nitro-arginine showed a certain effect on SHA, and it was also possible that these two drugs may play a role in inhibiting inflammatory factors.

## 5. Conclusion

In this research, a CVS model of subarachnoid hemorrhage was constructed in rats. Then, baicalein and L-nitro-arginine were adopted for intervention to analyze the mechanism of damage to peripheral blood cells and blood vessels in rats. The results showed that α-SMA was expressed in rat pericytes, and the expression of α-SMA in pericytes after L-nitro-arginine intervention was superior to that in the baicalein intervention group. After the action of the two drugs, the concentration of NO increased, and the response of vascular smooth muscle to NO increased significantly. In addition, the enzyme activity increased, so as to alleviate CVS after SAH. The innovation of this study is that the molecular mechanism of L-nitro-arginine and scutellarin and the changes of nitric oxide concentration in vivo are explored after the intervention of the cardiovascular system of rats after SAH. In conclusion, it is confirmed that both L-nitro-arginine and scutellarin have certain effects on neurovascular injury in SAH rats, and the effect of L-nitro-arginine is significantly superior to that of scutellarin.

## Highlights

1. The cerebral vasospasm model after SAH was successfully established.
2. L-nitro-arginine and scutellarin were used for intervention measures.
3. Changes of cerebral vasospasm mechanism of two drugs after SAH were measured.
